# Whole-Genome Shotgun Sequencing and Annotation of *Mycobacterium tuberculosis* MTBR1/09 Isolated from a Sputum Sample in Malaysia

**DOI:** 10.1128/genomeA.00513-16

**Published:** 2016-06-23

**Authors:** Rahizan Issa, Valentinus H. Seradja, Mohd Khairul Hafizi Abdullah, Hatijah Abdul

**Affiliations:** Bacteriology Unit, Infectious Disease Research Centre, Institute for Medical Research, Kuala Lumpur, Malaysia

## Abstract

This is a report of an annotated genome sequence of *Mycobacterium tuberculosis* MTBR1/09. The organism was isolated from a sputum sample from a male patient in Malaysia.

## GENOME ANNOUNCEMENT

In Malaysia, important successes have been achieved in tuberculosis (TB) control nationwide (1); however, they were in the early 2000s. At present, achieving TB control in places, such as Sabah, is a challenging task (2). In this report, the clinical isolate *Mycobacterium tuberculosis* MTBR1/09, obtained from a sputum sample, was subjected to whole-genome shotgun sequencing.

The total genomic DNA of *M. tuberculosis* MTBR1/09 was extracted using QIAxtractor and reagent packs (Qiagen, Düsseldorf, Germany), according to the manufacturer’s instruction. DNA concentrations were measured using a NanoVue Plus (GE Healthcare, Buckinghamshire, United Kingdom) spectrophotometer. The Illumina MiSeq platform was used for the whole-genome shotgun sequencing.

This resulted in 3,284,759 filtered reads with an average read length of 103.1 bp and approximately 61.5-fold coverage. The filtered reads were *de novo* assembled with CLC Genomics Workbench version 6.0.1, generating 203 contigs (*N*_50_, 62,347 bp). The genome contains a total of 4,358,280 bp, with a G+C content of 65.56%.

The NCBI Prokaryotic Genome Annotation Pipeline was performed. The genome was identified as having 4,177 genes, 3,933 coding sequences (CDSs), and 195 pseudogenes. Three types of rRNA (5S, 16S, and 23S) were annotated, and each has one rRNA. One noncoding RNA (ncRNA) and 45 tRNAs were annotated in this strain.

The spoligotype of *M. tuberculosis* MTBR1/09 was determined. Using the SpolPred software, it was determined to be 677777477413731, belonging to Indo-Oceanic lineage.

### Nucleotide sequence accession number.

This whole-genome shotgun project has been deposited in DDBJ/ENA/GenBank under the accession no. LATN00000000. The version described in this paper is the first version.
